# Maternity and Welfare Work

**Published:** 1920-07-10

**Authors:** 


**THE PUBLIC WELL-BEING**—(*continued*).

### Maternity and Welfare Work.

### Important Proposals in Birmingham.

The important and beneficial effects of maternity and child welfare work seem to be proved conclusively by the experience of the Birmingham Corporation, which has thirteen municipal centres in addition to eight voluntary concerns, and proposes further developments in this phase of civic activity. The Corporation's 1919 report shows that 19,335 babies were born during the year and that 87 per cent. of them were visited at their homes. Subjoined are results since Birmingham began to take a keen interest in welfare work; the figures being the infantile death-rates per 1,000 birth:—1905, 141; 1906, 157; 1907, 133; 1908, 130; 1909, 121; 1910, 115; 1911, 150; 1912, 111; 1913, 129; 1914, 120; 1915, 118; 1916, 104; 1917, 101; 1918, 99; 1919, 84.

The Corporation does its work thoroughly. It pays £1,400 a year to the Women's Hospital, so that fourteen beds may be allocated to puerperal fever cases, and has recently arranged with the Birmingham Maternity Hospital for the setting aside of twenty beds for difficult cases of pregnancy, the grant for this being £2,000 per annum. The Corporation is also arranging to deal with such babies as part of the Taplow Hospital which is being erected at Witton; and grants £500 a year to the Monument Road Day Nursery, which receives ten children whose mothers are undergoing hospital or sanatorium treatment.

Nevertheless, the Corporation realises that such good work needs extension. The medical and nursing services for maternity in Birmingham and elsewhere are obviously capable of great improvement. Of the 222 midwives in Birmingham in 1916 only seventy-five were "trained." During the five years 1913-17 twenty-eight cases of grave complaint against midwives have been reported to and investigated by the Public Health Committee, and of these twenty-two have been sent on to the Central Midwives Board. In most of these cases there are serious charges of ignorance or neglect, and not infrequently the life of mother or child has been at stake.

### Medical Men and Midwifery.

The medical officer of health declares that the medical service for assisting midwives in the 15,000 or 16,000 births, which they attend in normal times, is yearly becoming more insufficient by reason of the duties cast on medical men by the Insurance Act. A very large number of medical men have given up general midwifery. When asked to accept duty on the call of a midwife 210 medical men out of 320 declined to do so. The result is that constantly the complaint is made when a midwife sends for a doctor, as she is obliged to do under certain conditions, several are called on before one is obtained, and often hours of precious time are lost and unnecessary suffering endured. In one case the whole night was occupied in endeavours to obtain the services of a medical man, no less than seven being called upon before one was secured.

Hence he advocates a subsidised midwifery service, explaining that the present position is unsatisfactory in several respects. The Regulations of the Central Midwives Board allow midwives to continue practice until they voluntarily retire or commit such a serious offence as leads to their removal from the Midwives Roll. The midwife's earnings are often inadequate. If well-trained and capable women are to be attracted to the service a sufficient remuneration should be guaranteed, and to maintain a high standard of efficiency it is also necessary that provision for retiring allowances should be made, otherwise the ordinary midwife is under the necessity of continuing her profession until she becomes incapable on account of old age. At the present time many women trained in midwifery do not enter the profession on account of the difficulty of securing an adequate income. They prefer to obtain appointments as health visitors, welfare workers, or similar posts, where they can be assured of reasonably good and regular incomes. His suggestion is that the Corporation should subsidise approved midwives so as to ensure for each an income of £200 a year.

### Health Committee's Plans.

The report of the Corporation Health Committee makes some remarkably interesting recommendations. With regard to medical service it advocates arrangements which will secure that every woman after confinement shall have the opportunity of consulting a medical practitioner at least once. It is assured that it is possible for a woman to suffer all her life from ailments which might have been avoided if medical care had been available at and after her confinement. It therefore proposes to appoint medical officers to undertake this work, in cases in which a private practitioner is not employed. The services of three whole-time officers would be required, and the total cost would ultimately be about £3,000 per annum.

The Committee also feels it desirable to establish a certain number of Midwifery Homes, where mothers can go for confinement, and where some of

THE PUBLIC WELL-BEING—(*continued*)

the approved midwives can reside. Action in this direction should be taken gradually, and the Committee suggests the establishment of ten homes, each to have accommodation for a midwife, two pupils, and six or eight cases of confinement. This provision would help to meet the great need that exists for more suitable places for confinement than are available in many of the courtyard dwellings in the City. It is estimated that each of these homes would cost about £1,300 to furnish and equip, and that the annual cost, taking an average of five patients, would be about £720 per home, reckoning the cost of each bed at three guineas for a period of fifty weeks per year, and the income at an average of 15s. per week, and putting down the sum of £120 for rent, etc. The capital expenditure on the ten homes would thus amount to £13,000, and the annual cost for rent, etc., £7,200.

The Committee also recommends a guaranteed salary of £120 a year each for approved maternity nurses, makes provision for the services of home helps at a considerable expenditure, and deals with other phases of the problem in a bold and public-spirited manner. The development of the Birmingham proposals should be watched with keen interest by all the other great municipal bodies in the Kingdom.

